# Effect of Aging Temperature on the Microstructure and Mechanical Properties of a Novel β Titanium Alloy

**DOI:** 10.3390/ma16237393

**Published:** 2023-11-28

**Authors:** Wei Xiang, Wuhua Yuan, Hao Deng, Hengjun Luo, Longqing Chen, Weidong Yin

**Affiliations:** 1 College of Materials Science and Engineering, Hunan University, Changsha 410082, China; xiangwei03300121@163.com (W.X.); luohengjun2022@163.com (H.L.); 2 China National Erzhong Group Deyang Wanhang Die Forging Co., Ltd., Deyang 618013, China; denghaoscu@126.com (H.D.); 18781088843@163.com (W.Y.); 3 Key Laboratory of Radiation Physics and Technology of Ministry of Education, Institute of Nuclear Science and Technology, Sichuan University, Chengdu 610064, China; chenlongqing@scu.edu.cn

**Keywords:** metastable β titanium alloys, aging temperature, microstructure, mechanical properties

## Abstract

High-strength metastable β titanium alloys are promising structural materials to be used in aviation industries. In order to achieve a high strength level, solid solution treatment within β region and subsequent low-temperature aging are usually necessary to obtain fine α precipitates. The selection of the aging temperature is considered critical to the mechanical performance of metastable β titanium alloys. In this work, we investigated the effect of aging temperature on the microscopic structure and mechanical properties of a novel type of titanium alloy TB18 (Ti-4.5Al-5Mo-5V-6Cr-1Nb). A series of aging treatments were conducted on TB18 specimens at 510 °C, 520 °C, 530 °C, and 540 °C after the solid solution treatment at 870 °C. On the basis of the systematic results of scanning electron microscope and transmission electron microscope, the behavior of the α phases affected by the varied aging temperatures were studied. As the aging temperature rose, the grain width of the α phase increased from 60 nm (510 °C) to 140 nm (540 °C). For the TB18 samples aged at 510 °C and 540 °C, the tensile strength/yield strength/impact toughness values were 1365 ± 3 MPa/1260 ± 0.9 MPa/26.5 ± 1.2 J/cm^2^ and 1240 ± 0.9 MPa/1138 ± 0.8 MPa/36.2 ± 1.3 J/cm^2^, respectively. As a result, the tensile performance and the grain width of the α phase agreed well with the Hall–Petch relationship. This work offers valuable support for both theoretical analyses and the heat treatment strategies on the novel TB18 titanium alloy.

## 1. Introduction

Titanium alloys have gained immense popularity in various critical engineering applications due to their exceptional combination of properties, including high strength-to-weight ratio [1,2], corrosion resistance [3,4], and heat tolerance [5]. Among these alloys, Ti-4.5Al-5Mo-5V-6Cr-1Nb, also known as TB18, stands out as a prime candidate for use in aerospace, gas turbine engines, and other high-performance applications [6]. Its unique composition, featuring aluminum, molybdenum, vanadium, chromium, and niobium, offers a versatile platform for engineering materials that can withstand harsh environmental conditions and elevated temperatures [7,8,9].

It is widely acknowledged that the mechanical characteristics of near-beta titanium alloys are closely tied to their microstructures [10]. A fine equiaxed primary α (α_p_) structure can significantly enhance properties like strength, hardness, ductility, and toughness. The precipitation of α_s_ phase within the β matrix can bolster tensile strength, with a reduction in size or an increase in volume fraction contributing to improved yield strength. Additionally, the size of prior β grains and the morphology of the α_p_ phase can influence ductility [11,12]. It is important to note that the microstructure is primarily determined by the heat treatment process. Therefore, gaining a deep understanding of the intricate connections between heat treatment, microstructure, and mechanical properties is vital for material engineers and designers to optimize their heat treatment strategies and control microstructural outcomes [13,14,15].

Before this point, numerous pieces of literature have explored the connection between heat treatment procedures and their impact on microstructures and mechanical properties. Li et al. [16] found that when TB17 alloy undergoes a solution treatment at 805 °C (i.e., below the phase transition point), followed by aging, it can attain an optimal balance of high strength at 1375 MPa and noteworthy ductility. This achievement is attributed to the mixed microstructure that includes an appropriate quantity of microscale α and nanoscale α_s_ precipitates. Wu et al. [17] conducted research on the interplay between the microstructure and mechanical characteristics of Ti55531 alloy during heat treatment. Their findings revealed that the α_p_ grain size, shape, and volume are predominantly determined by the solution temperature, while factors like the width, length, morphology, orientation, and nucleation site of α_s_ grains are under the sway of the aging temperature and duration. In their study, Dong et al. [18] observed that Ti-7333 alloy aged at 520 °C resulted in a higher density of precipitates and finer α plates compared to the alloy aged at 500 °C. This outcome is beneficial for enhancing strength. In summary, heat treatment is essential for controlling the mechanical properties of titanium alloys and is a crucial step in the manufacturing of titanium alloys. Nonetheless, research on the heat treatment of TB18 titanium alloy remains insufficient, and there exists a lack of clarity regarding the evolution of microstructure and mechanical properties throughout the heat treatment process. This limitation hinders the production and application of TB18 alloy. Hence, there is an immediate need to investigate the impact of heat treatment on the microstructure and mechanical properties of TB18 titanium alloy [19].

In this study, TB18 alloy was treated with solution above the phase transition point and then further aged to obtain ultrahigh strength lamellar structure. The objective of this study was to examine how aging temperature influences the microstructure and mechanical characteristics of TB18 alloy, investigate the plastic deformation and fracture mechanisms within the TB18-layered structure, and establish the relevant connections between microstructure and mechanical properties.

## 2. Experiment Section

### 2.1. Materials and Experimental Procedures

The TB18 alloy billet used in the present work was provided by Wes (Xi’an, China). The chemical composition of the TB18 billet was determined to be Ti-4.4Al-5.1Mo-4.9V-5.8Cr-1.2Nb-0.15Fe-0.15O-0.05N-0.08C-0.015H (wt.%). The β-transus temperature was 800 °C, which was measured with the metallographic method.

The TB18 titanium alloy initially underwent a forging process within the α + β phase zone at 770 °C. Subsequently, it was subjected to a solid solution heat treatment at 870 °C for 2 h, followed by air cooling. To investigate how the microstructure and mechanical properties of the TB18 titanium alloy were influenced by the aging temperature, aging was carried out at temperatures of 510 °C, 520 °C, 530 °C, and 540 °C, each for a duration of 4 h.

### 2.2. Preparation of the Samples

Microstructural analyses were conducted through various techniques, including optical microscopy (OM, DMi8 M/C/A, Ernst Leitz Company, Wetzlar, Germany), scanning electron microscopy (SEM) using an Inspect F50 instrument from FEI (Hillsboro, OR, USA), with electron backscatter diffraction (EBSD) capabilities provided by a NordlysNano system from Oxford Instruments (Oxford, UK), and transmission electron microscopy (TEM) utilizing an F20 apparatus from FEI.

For the OM and SEM examinations, the samples were meticulously ground with sandpaper (SiC #220 to SiC #5000) and polished with a silica suspension (0.06 μm) until the samples’ surfaces became specular. Subsequently, they were etched with Kroll’s agent, which consists of 1 vol% HF, 4 vol% HNO_3_, and 95 vol% H_2_O. The TEM samples were prepared by employing ion milling, specifically utilizing a PIPS II 695 system from Gatan Inc. (Santiago, Chile). As for the EBSD analysis, the samples were polished using a vibration polisher, specifically the Buehler VibroMet 2 (Lake Bluff, IL, USA), for stress relief and then taken for EBSD testing (an accelerating voltage of 20 kV, a step size of 4 μm, and a sample tilt angle of 70°. The data generated from EBSD were processed using Channel 5 software (v.15.5.0).

Tensile testing was performed using an Instron 8801 machine, which was equipped with an extensometer to accurately measure the strain. Standard M10 cylindrical tension test specimens were prepared in accordance with the ASTM E8/E8M-16a standard [20]. The tensile tests were conducted at a constant strain rate of 0.5 mm/min. To obtain representative values, three individual tensile testing specimens were examined to determine the average values of the ultimate tensile strength (UTS), yield strength (YS), and elongation (El).

U-notch impact samples were prepared by cutting. Subsequently, we conducted room-temperature impact tests using the NI300C oscillographic impact testing machine (The NCS Testing Technology Co., Ltd., Beijing, China). To ensure the precision of our experiments, we selected three samples for each condition and calculated the average values from the results.

## 3. Results and Discussion

**Figure 1:**
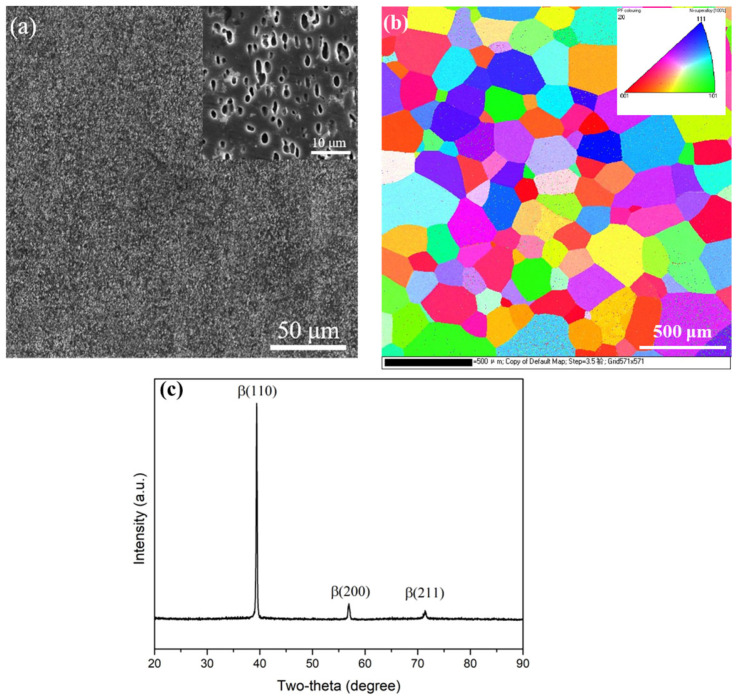
(**a**) Microstructure of a TB18 raw bar after two-phase zone hot deformation; (**b**) electron backscatter diffraction results of β grains in the sample obtained by water quenching after solid solution treatment above the phase transition point; (**c**) X-ray diffraction results of the sample obtained by water quenching after solid solution treatment above the phase transition point.

Figure 1a shows the microstructure of a TB18 raw bar after two-phase zone hot deformation. Flattened β grains are observed along the direction of the compressive axis, and serrated or discontinuous β grain boundaries can also be observed in the alloy microstructure. A large number of equiaxed α particles are uniformly distributed in the matrix, indicating that the α phase has undergone obvious spheroidization after deformation [21,22]. Figure 1b shows the electron backscatter diffraction (EBSD) results of β grains in the sample obtained by water quenching after solid solution treatment above the phase transition point. After being annealed at 870 °C for two hours, the original β deformed structure underwent complete recrystallization, forming a uniform equiaxed β structure, and the EBSD results showed no obvious texture of β grains after solid solution treatment. Figure 1c shows the X-ray diffraction (XRD) results of the sample obtained by water quenching after solid solution treatment above the phase transition point. The XRD results show that the organization obtained by water quenching after solid solution treatment at 870 °C all consists of the β phase without ω-phase or α-phase precipitation. This is because the β-stabilizing elements in the TB18 alloy are high, and under the conditions of rapid cooling, it is easy to stabilize the β phase at room temperature and prevent it from transforming to other phases [23].

**Figure 2:**
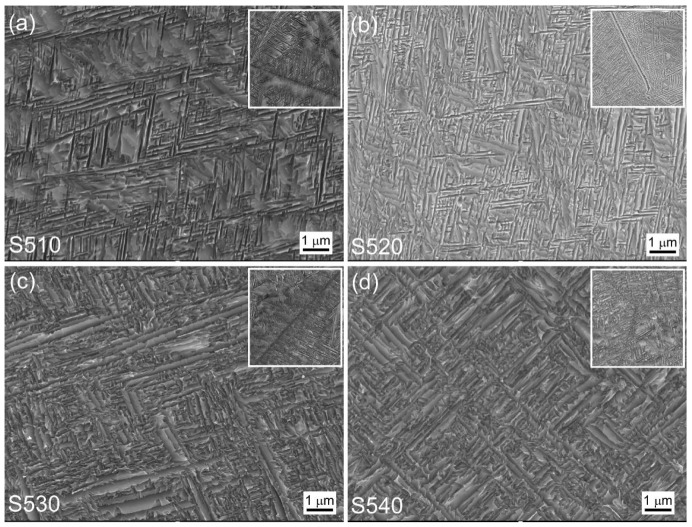
SEM images of the samples at different aging temperatures: (**a**) 510 °C; (**b**) 520 °C; (**c**) 530 °C; (**d**) 540 °C.

Figure 2a–d show SEM images of four samples (named S510, S520, S530, and S540) after 5 h of aging at 510 °C, 520 °C, 530 °C, and 540 °C, respectively, following solid solution treatment. The images reveal that all four samples exhibited similar microstructures. Because of the 2 h solid solution treatment above the phase transition point, there was no restriction from α grain boundaries, resulting in the rapid growth of the original β grains. As a result, all four samples formed coarse β grains [24]. During the solid solution stage above the phase transition point, the original β grains underwent sufficient recovery and recrystallization, and the grain boundaries became straight and continuous instead of being curved and discontinuous. During the subsequent cooling process and aging stage, the α phase preferentially precipitated along the grain boundaries, resulting in straight and continuous grain boundaries observed in the magnified images of the upper right corner of all four samples. Additionally, because of the fast cooling rate, the α phase at the grain boundaries was relatively fine, and no obvious nonprecipitation zone was observed near the grain boundaries [16].

**Figure 3:**
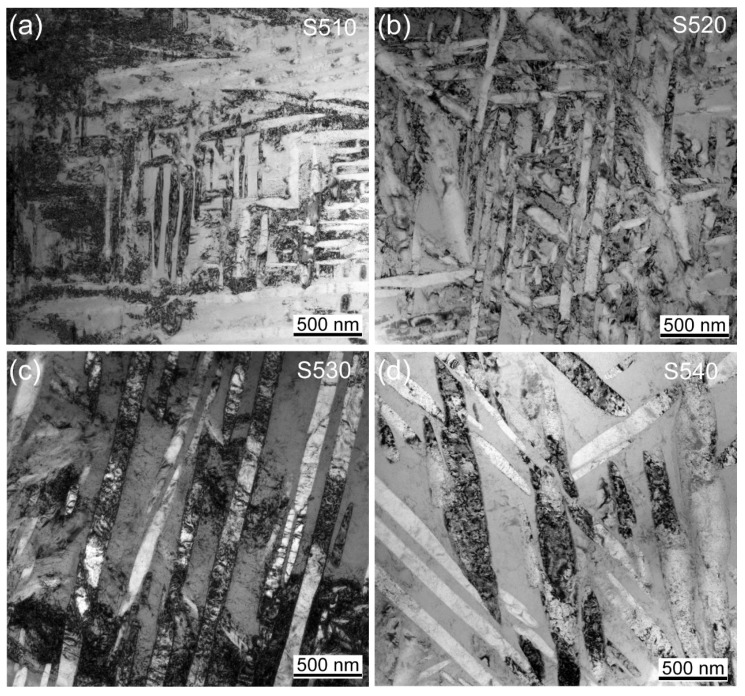
TEM images of samples at different aging temperatures: (**a**) 510 °C; (**b**) 520 °C; (**c**) 530 °C; (**d**) 540 °C.

During the aging stage, the α phase precipitated uniformly and interlaced with each other inside the grains, and small and densely distributed α precipitates were observed in all four samples. Figure 2a shows the very small size of the α-phase precipitates at an aging temperature of 510 °C. Further characterization of the microstructure of the α precipitates was performed using TEM, as shown in Figure 3a. The average grain width of the precipitates was only 60 nm, and the corresponding results are listed in Table 1. The precipitated α phase had a relatively uniform size distribution and a high number density.

The microstructural changes in Figure 2b–d indicate an increasing trend in the size of the α-phase precipitates inside the grains with the increasing aging temperature. The corresponding TEM results are shown in Figure 3b–d, and the statistical results show that the average grain width of the α-phase precipitates inside the grains increased from 81 nm at an aging temperature of 520 °C to 102 nm at 530 °C and significantly increased to 140 nm at 540 °C. The statistical results are listed in Table 1.

Moreover, as the temperature increased, the size distribution of the precipitated α phase became uneven, and the number density of the α precipitates decreased. This is because the aging temperature caused the growth of the α phase, and the coarsening of this layered α phase can be explained by the migration of solute atoms. The difference in the curvature at the α/β-phase interface resulted in a difference in potential energy. The curvature at the end and defect positions of the layered α phase was larger, resulting in a higher potential energy, while the curvature at the smooth position was smaller, resulting in lower potential energy. Therefore, solute atoms migrated from the end or defect positions of the layered structure with higher potential energy to the smoother positions with lower potential energy, which is known as the end-migration mechanism [25].

The end-migration mechanism causes the end of the layered structure to gradually disappear, while the smooth part gradually becomes thicker, resulting in the coarsening of the α phase. Increasing the aging temperature increases the atomic diffusion rate and the grain boundary migration rate, resulting in a significant increase in the size of the α phase. On the other hand, increasing the aging temperature reduces the precipitation rate of the α phase. Therefore, at lower aging temperatures, the α phase reaches saturation after a short time of precipitation, and the rate of coarsening of the α phase is slow. When the first α-phase precipitates have not grown significantly, the rest of the α-phase precipitates also precipitate successively, resulting in a uniform α phase size. However, at higher aging temperatures, the precipitation rate of the α phase is slow, and the coarsening rate of the α phase is fast. When the first α-phase precipitates have grown significantly, the rest of the α-phase precipitates have just precipitated, resulting in an uneven size distribution of the α phase. Moreover, during the aging process, the recovery of the matrix and the precipitation of the α phase occur simultaneously. As the aging temperature increases, the precipitation rate of the α phase gradually becomes slower than the recovery rate of the matrix. Therefore, many dislocations and defects recover and cannot become nucleation points for the α phase [26]. As a result, the precipitation of the layered α phase decreases, and the spacing between the layered α phases gradually increases, resulting in a decrease in the number density.

**Figure 4:**
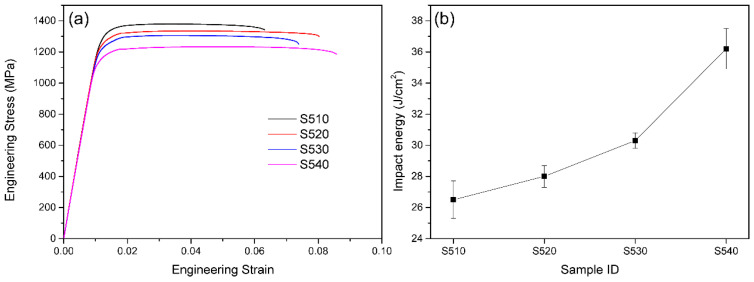
Mechanical properties: (**a**) the room temperature engineering stress–strain curves of TB18 alloy samples treated at different aging temperatures; (**b**) the impact toughness of TB18 alloy samples treated at different aging temperatures.

Figure 4a shows the room temperature engineering stress–strain curves of TB18 alloy samples treated at different aging temperatures. As shown in the figure, with the increase in aging temperature, the strength of the alloy decreases, while the elongation overall increases. However, the elongation of the sample aged at 520 °C is greater than that of the sample aged at 530 °C. This is because the deformation mechanism of the layered TB18 alloy is mainly shear deformation and dislocation slip. The coarse layered α phase (at higher aging temperatures) can provide a longer length for dislocation slip, resulting in lower strength and better plasticity.

In contrast, the fine α-phase precipitates that precipitate at lower aging temperatures have a large number of α/β interfaces, which can hinder dislocation slip, resulting in higher strength and lower plasticity [27]. Therefore, as the aging temperature increases, the size of the α phase increases, and the number density decreases, resulting in a decrease in the strength of the alloy and an increase in plasticity. However, since all samples aged at different temperatures have continuous grain boundary α, the area near the grain boundary α becomes softer. Therefore, cracks are prone to nucleate near the grain boundary, and once a crack nucleates, it will rapidly propagate along the grain boundary, leading to premature failure of the sample [28]. Therefore, anomalies in the plasticity change trend of the samples aged at 520 °C and 530 °C occurred. The tensile mechanical properties of the TB18 alloy were obtained from the stress–strain curves under different aging conditions. All data were obtained by averaging the results of three sets of samples and calculating the standard deviation, as shown in Table 1.

The samples treated with aging at 510 °C have the highest ultimate tensile strength and yield strength of 1365 MPa and 1260 MPa, respectively, but the lowest elongation of only 6.5%. The samples treated with aging at 520 °C have a lower ultimate tensile strength and yield strength of 1339 MPa and 1235 MPa, respectively, but the elongation increases to 7.8%. Compared with the samples treated with aging at 520 °C, the samples treated with aging at 530 °C have slightly lower ultimate tensile strength and yield strength of 1305 MPa and 1215 MPa, respectively, and the elongation also decreases to 7%. The samples treated with aging at 540 °C have the lowest ultimate tensile strength and yield strength of 1240 MPa and 1138 MPa, respectively, but the highest elongation of 10.5%.

Figure 4b shows the impact toughness of TB18 alloy samples after different aging temperature treatments. As can be seen from the figure, with the increase in aging temperature, the impact energy required for the sample to fracture increases. Detailed results are shown in Table 1, where it can be observed that as the aging temperature increases from 510 °C to 540 °C, the impact energy of the alloy increases from 26.5 J/cm^2^ to 36.2 J/cm^2^. As can be inferred from the above analysis, with the increase in aging temperature, the precipitated α phase in the alloy will gradually coarsen. According to the Hall–Petch relationship, at lower aging temperatures, the finely layered α-phase precipitates in the alloy have high strength and cannot undergo effective deformation. When cracks propagate, they will bypass the harder finely layered α precipitates. At higher aging temperatures, the coarser layered α-phase precipitates in the alloy are softer compared to the finer layered α-phase precipitates, and can undergo effective deformation. When cracks propagate, the cracks can pass through the coarser layered α-phase precipitates, resulting in a more tortuous crack propagation path during deformation of the alloy. Although the initiation energy of cracks will decrease to some extent with the increase in aging temperature, the propagation energy of cracks will significantly increase, and the resistance to crack propagation will be significantly enhanced. Therefore, the impact energy required for the alloy to fracture will be higher. This indicates that the alloy’s toughness is improved at higher aging temperatures. The results suggest that controlling the aging temperature can effectively regulate the microstructure and mechanical properties of the TB18 alloy. These findings are important for understanding the relationship between microstructure and mechanical properties in TB18 alloys and for optimizing the aging process of these alloys for various applications. Further research is needed to fully understand the underlying mechanisms of the observed changes in microstructure and mechanical properties with aging temperature.

As is well known, the mechanical properties of a material are determined by its microstructure. The relationship between the yield strength of an alloy and the grain width of the α phase within its crystal structure can be mathematically described using the Hall–Petch relationship. This relationship is an important tool for understanding the mechanical behavior of alloys and can be used to optimize their performance [29,30].

(1) $$\sigma_{y}=\sigma_{0}+kd^{-1/2}$$

where *σ_y_* represents the yield strength of the alloy, while *d* refers to the average grain width of the α phase within its crystal structure. *σ*_0_ and *k* are material constants. The Hall–Petch relationship demonstrates that the yield strength of an alloy increases as the grain width of the α phase within its crystal structure decreases. This relationship is an important tool for understanding the mechanical behavior of alloys and can be used to optimize their performance.

**Figure 5:**
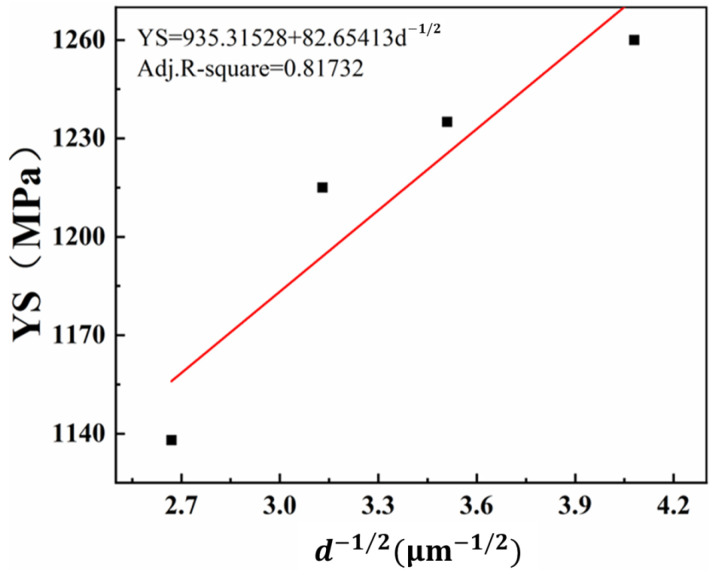
Hall–Petch relationships for the yield strength and the grain width of the lamella α-phase in TB18 alloys.

In order to understand the relationship between the grain width of the α phase within the crystal structure and the yield strength of alloys, the average grain width of the α phase at different aging temperatures was statistically analyzed and fitted with the corresponding yield strength of the alloy. It was found that the fitting results were in accordance with the Hall–Petch relationship. Figure 5 shows the relationship between the yield strength of the alloy and the grain width of the α phase within its crystal structure at different aging temperatures. As the aging temperature increases, the grain width of the α phase within the crystal structure increases, while the yield strength of the alloy decreases. However, the relationship between the yield strength and *d*^−1/2^ in TB18 alloy is not entirely linear, as the volume fraction and number density of the α phase within the crystal structure also contribute to the yield strength of the alloy. These factors should also be taken into account when examining the mechanical behavior of the alloy.

Since the deformation mechanism has a significant impact on the mechanical properties of alloys, it is necessary to study the plastic deformation mechanism of alloys. In order to investigate the plastic deformation mechanism of the TB18 lamellar microstructure during the tensile fracture process, we conducted a TEM analysis of the microstructure in the plastic deformation zone of the S540 tensile sample.

**Figure 6:**
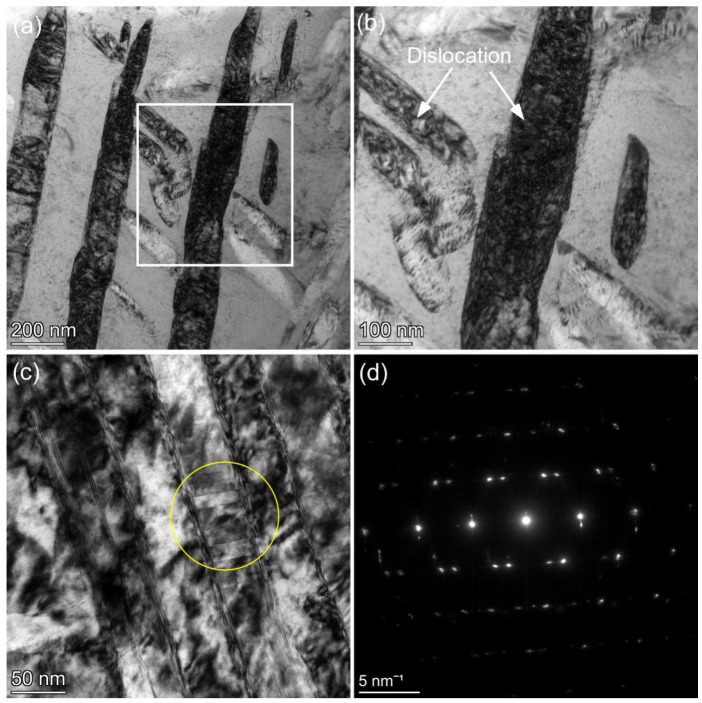
The microstructure of the plastic deformation zone of the S540 tensile sample, where (**a**) the plastic deformation zone of the S540 tensile sample, (**b**,**c**) the high magnification of the white boxed portion in (**a**,**d**) the corresponding selected area electron diffraction of the circled area of (**c**).

Figure 6a shows the microstructure of the plastic deformation zone of the S540 tensile sample, which is consistent with previous research results. The coarse lamellar α phase undergoes severe deformation, while the smaller α phase undergoes slight deformation, partly due to its higher hardness. Figure 6b depicts an enlarged view of the white box in Figure 6a, which shows a large number of dislocations in both the coarse and small lamellar α phases. Moreover, it is evident that the coarse lamellar α phase has a darker color, which is a characteristic of high dislocation density.

As mentioned in the previous analysis, coarse α-phase layers are more favorable for slip of dislocations and are prone to deformation. During the deformation process of coarse α-phase layers, dislocation slip, dislocation intersection, and dislocation entanglement occur, and eventually fracture occurs under the action of shear force. In Figure 6b, it can be clearly seen that the coarse α-phase layer is elongated along the length direction and shrinks along the width direction, and, finally, it is cut along the slip direction (45° to the length direction) [31].

Figure 6c depicts a microstructural image that further magnifies the white box in Figure 6a, which shows that deformation twinning occurs in the TB18 alloy layer structure during tensile deformation. A diffraction analysis of the twinning region is shown in Figure 6d. However, previous studies have shown that such deformation twinning is difficult to achieve in the α-phase layers and is usually observed in the equiaxed primary α deformation process. The observation of deformation twinning in the α-phase layers in this study was due to the coarsening of the α-phase layers with the increasing aging temperature. At the same time, the formation of deformation twinning indicates that larger shear stress is required for the TB18 layer structure’s deformation, and more slip systems will be activated during the deformation process, which can simultaneously improve the strength and plasticity of the alloy. In summary, the main deformation mechanisms of the TB18 alloy layer structure are slip, shear, and twinning deformation in the α-phase layers [32].

**Figure 7:**
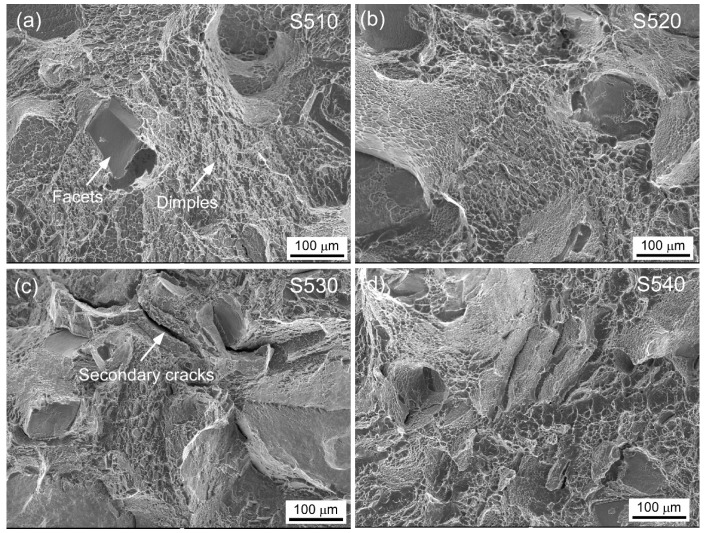
Fracture morphologies of samples at different aging temperatures: (**a**) 510 °C; (**b**) 520 °C; (**c**) 530 °C; (**d**) 540 °C.

In order to investigate the fracture mechanism of the TB18 alloy layer structure during tensile deformation, we characterized the SEM images of the tensile fracture surfaces of the samples at different aging temperatures. The SEM images of the tensile fracture surfaces of the samples at different aging temperatures (Figure 7a–d) all exhibit a mixed fracture mode of brittle fracture along the grain boundaries and ductile fracture with microvoid coalescence. Figure 7a shows the tensile fracture surface of the sample aged at 510 °C, where smooth cleavage planes and shallow and small dimples can be observed [33].

As the aging temperature increases, the proportion of smooth cleavage planes in the tensile fracture surface of the sample aged at 520 °C (Figure 7b) is significantly reduced, and the dimples become deeper and larger, indicating that the alloy has undergone greater deformation and reflects the improvement of the alloy’s plasticity. As the aging temperature increases to 530 °C, the proportion of cleavage planes in the alloy’s tensile fracture surface (Figure 7c) increases to a certain extent, but secondary cracks are observed. The appearance of secondary cracks increases the threshold energy for crack propagation, which is advantageous for improving plasticity [34]. However, compared with samples aged at 520 °C, the abundant occurrence of transgranular fracture still leads to a decrease in plasticity. When the aging temperature is further increased to 540 °C, the proportion of smooth cleavage planes in the alloy’s tensile fracture surface (Figure 7d) is already very low, and the dimples become larger and deeper, and a large number of steps are observed, which are the result of crack deflection during the propagation process, indicating that the tortuous crack propagation path of the alloy is beneficial to improving plasticity and, thus, exhibits the highest plasticity [35].

## 4. Conclusions

In this paper, the effects of different aging temperatures on the microstructure of TB18 alloy were studied in depth, and the fracture mechanism was discussed. On the basis of the results of this study, the main conclusions can be drawn as follows. 

As the aging temperature rose, the grain width of the α phase increased from 60 nm (510 °C) to 140 nm (540 °C).

The decrease in the aging temperature leads to the thinning of lamella α in TB18 alloy, which increases the strength of TB18 alloy and decreases the plasticity and impact toughness.

The tensile performance and the grain width of the α phase agreed well with the Hall–Petch relationship.

## Figures and Tables

**Table 1:** Lamellar α width and mechanical properties (UTS, YS, El and impact energy) of samples at different aging temperatures.

Aging Temperature	Width of α Lamella (nm)	UTS (MPa)	YS (MPa)	El (%)	Impact Energy (J/cm^2^)
510 °C	60	1365 ± 3	1260 ± 0.9	6.5 ± 0.1	26.5 ± 1.2
520 °C	81	1339 ± 1.7	1235 ± 1.2	7.0 ± 0.2	28.0 ± 0.7
530 °C	102	1305 ± 0.8	1215 ± 1.5	7.8 ± 0.1	30.3 ± 0.5
540 °C	140	1240 ± 0.9	1138 ± 0.8	10.5 ± 0.3	36.2 ± 1.3

## Data Availability

The authors confirm that the data supporting the findings of this study are available within the article.
